# Supplementary material for: Allelic Diversification of the Wx and ALK Loci in Indica Restorer Lines and Their Utilisation in Hybrid Rice Breeding in China over the Last 50 Years
Source: Int J Mol Sci. 2022 May 25;23(11):5941. doi: 10.3390/ijms23115941 (PMC9180661; doi:10.3390/ijms23115941)
Supplement: Supplementary file 1 [file ijms-23-05941-s001.zip › Supplementary figures and tables.pdf]

Supplementary Figures

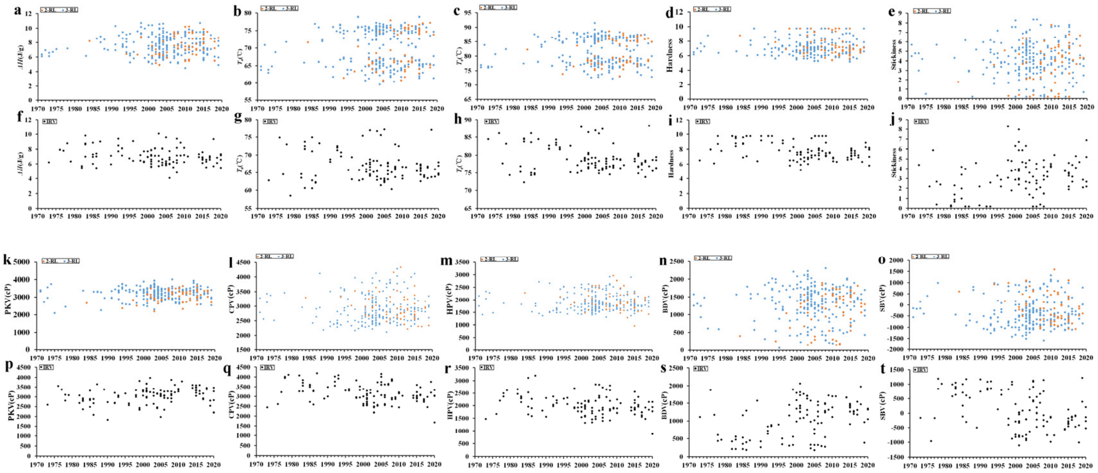

Figure S1. Phenotypic data for rice breeding distributions. **(a, b, c, d, e, k, l, m, n, o)** 2-RL and 3-RLs. **(f, g, h, i, j, p, q, r, s, t)** IRVs. **(a, f)** Enthalpy of gelatinisation. **(b, g)** Onset temperature of gelatinisation. **(c, h)** Terminating temperature of gelatinisation. **(d, i)** Hardness. **(e, j)** Stickiness. **(k, p)** Peak paste viscosity. **(l, q)** Cool paste viscosity. **(m, r)** Hot paste viscosity. **(n, s)** Breakdown value. **(n, t)** Setback value.

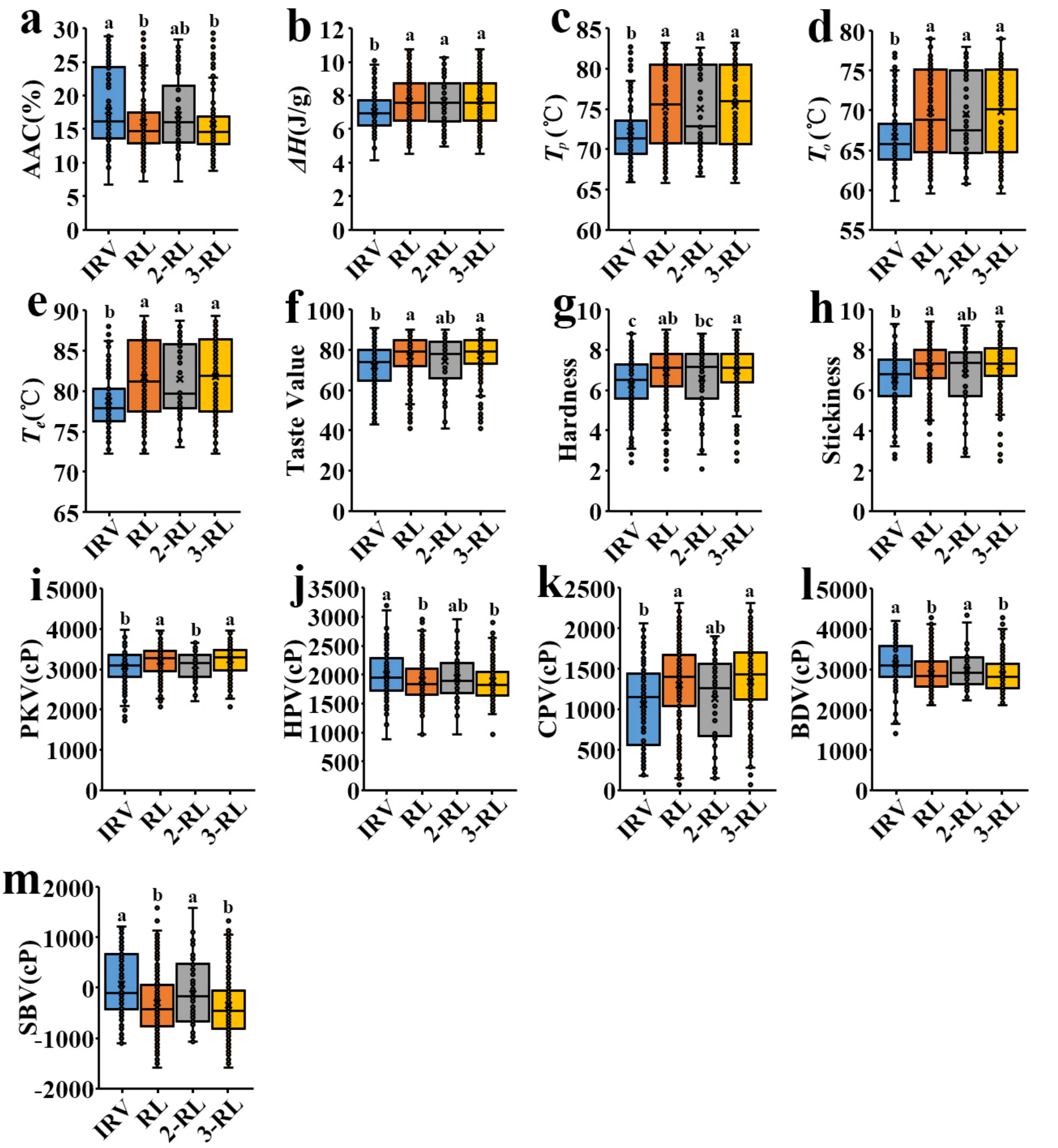

Figure S2. Phenotypic data for IRVs, 2-RLs and 3-RLs. **(a)** Apparent amylose content. **(b)** Enthalpy of gelatinisation .**(c)** Peak temperature of gelatinisation. **(d)** Onset temperature of gelatinisation. **(e)** Terminating temperature of gelatinisation. **(f)** Taste value. **(g)** Hardness. **(h)** Stickiness. **(i)** Peak paste viscosity. **(j)** Hot paste viscosity. **(k)** Cool paste viscosity. **(l)** Breakdown value. **(m)** Setback value. One-way ANOVA, different letters represent significant differences (p <0.05).

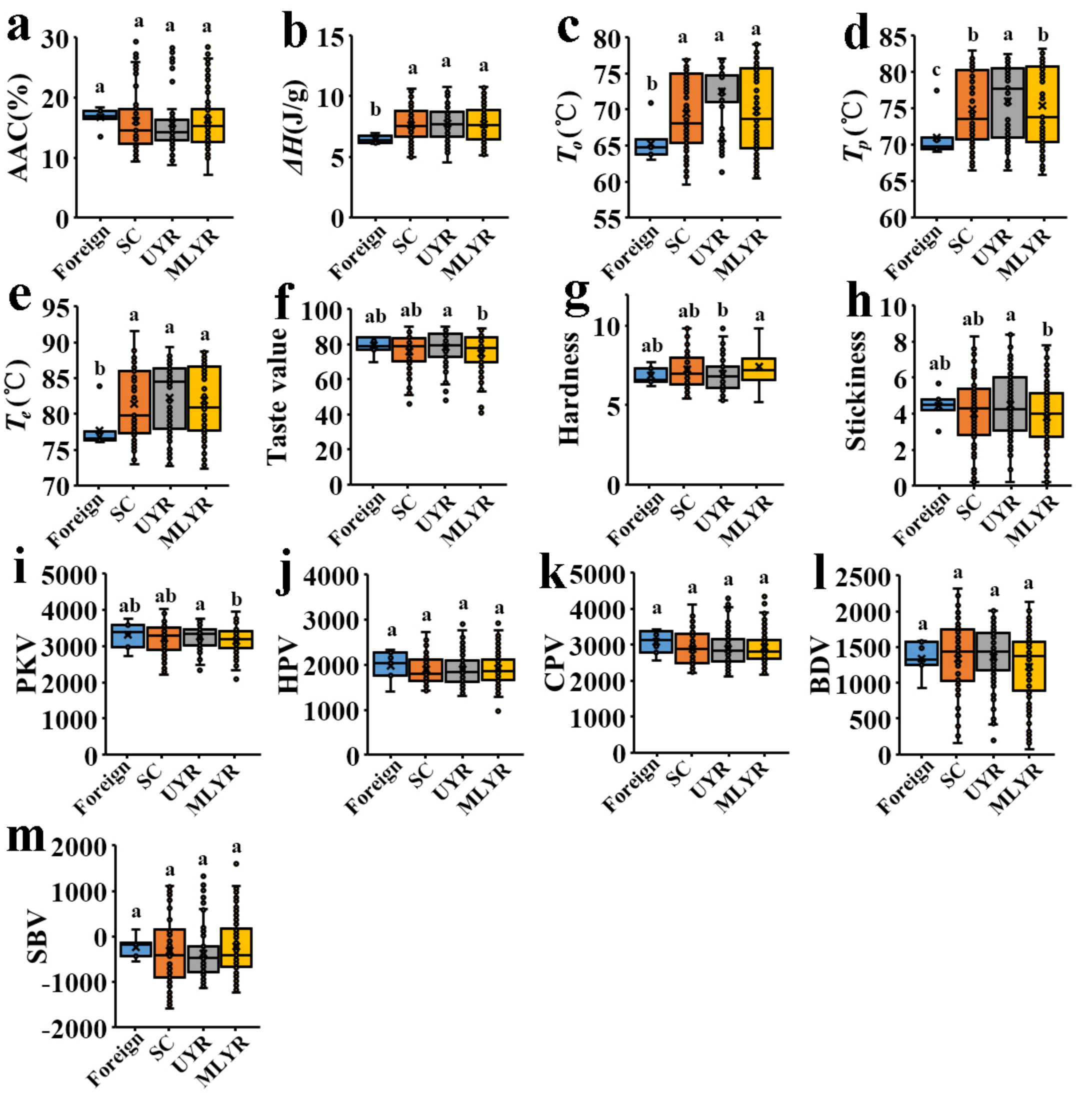

Figure S3. Phenotypic data for different areas. **(a)** Apparent amylose content. **(b)** Enthalpy of gelatinisation **(c)** Peak temperature of gelatinisation. **(d)** Onset temperature of gelatinisation. **(e)** Terminating temperature of gelatinisation. **(f)** Taste value. **(g)** Hardness. **(h)** Stickiness. **(i)** Peak paste viscosity. **(j)** Hot paste viscosity. **(k)** Cool paste viscosity. **(l)** Breakdown value. **(m)** Setback value. One-way ANOVA, different letters represent significant differences (p <0.05).

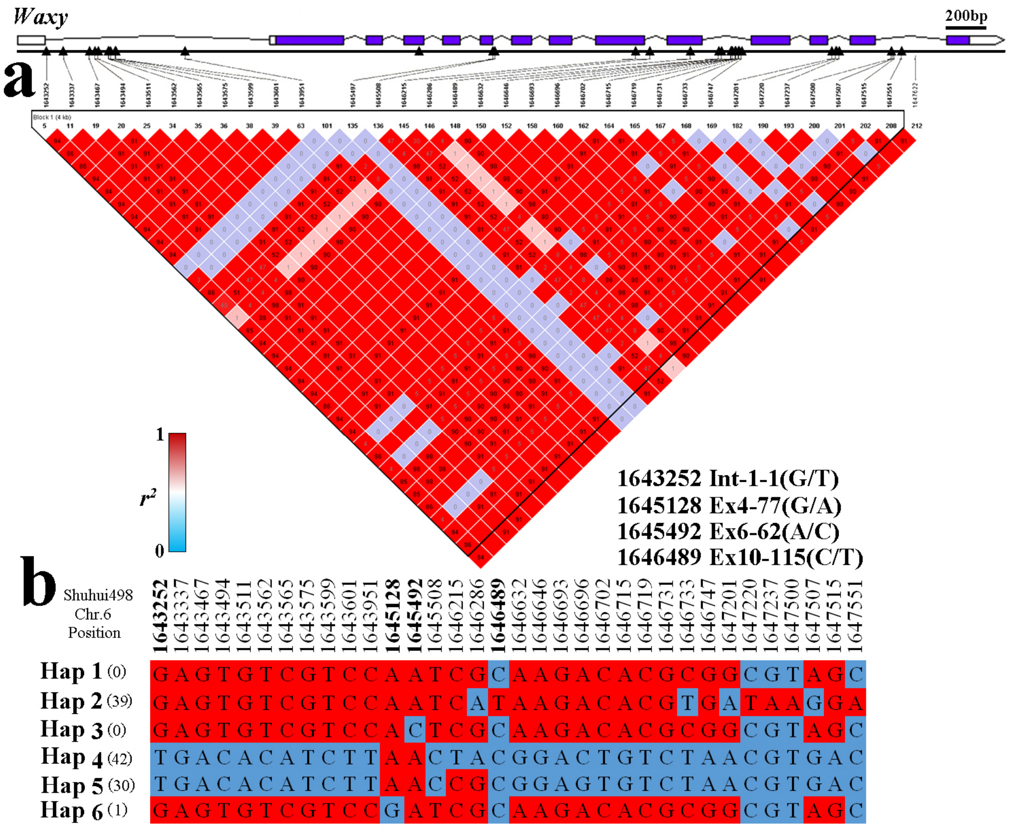

Figure S4. Analysis of haplotypes of *Waxy* in IRVs. **(a)** Linkage disequilibrium (LD) analysis. The r^2 value is shown on the matrix diagram. **(b)** Haplotype analysis.

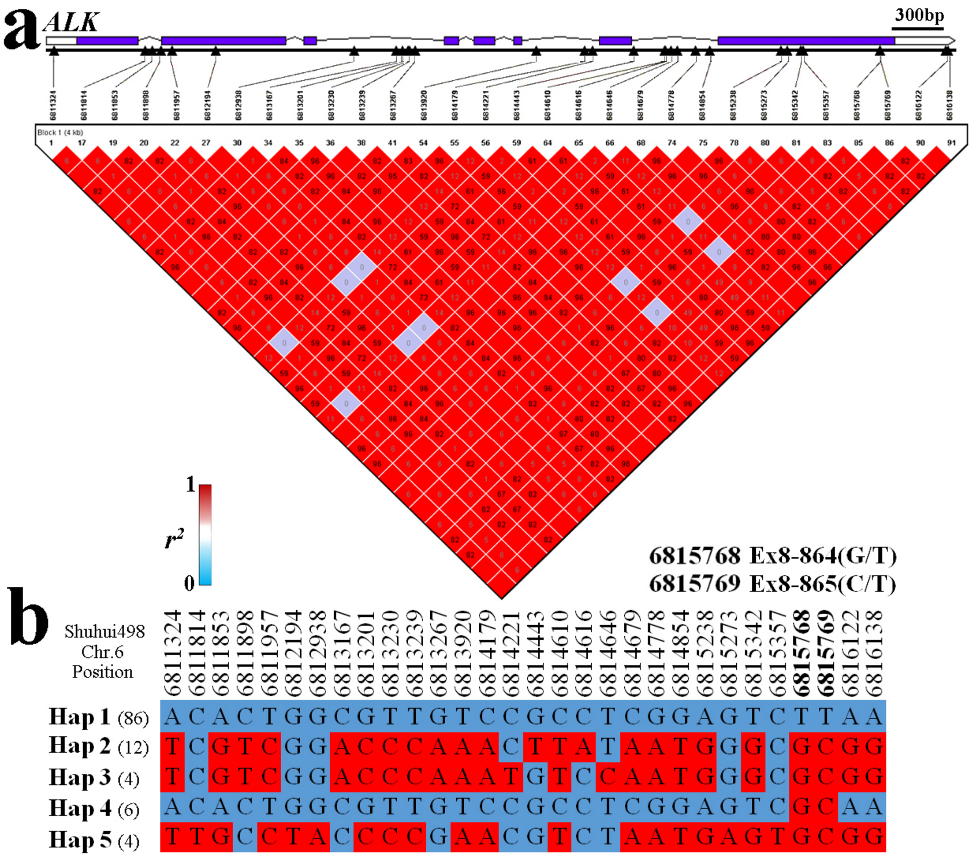

Figure S5. Analysis of haplotypes of *ALK* in IRVs. **(a)** Linkage disequilibrium (LD) analysis. The r^2 value is shown on the matrix diagram. **(b)** Haplotype analysis.

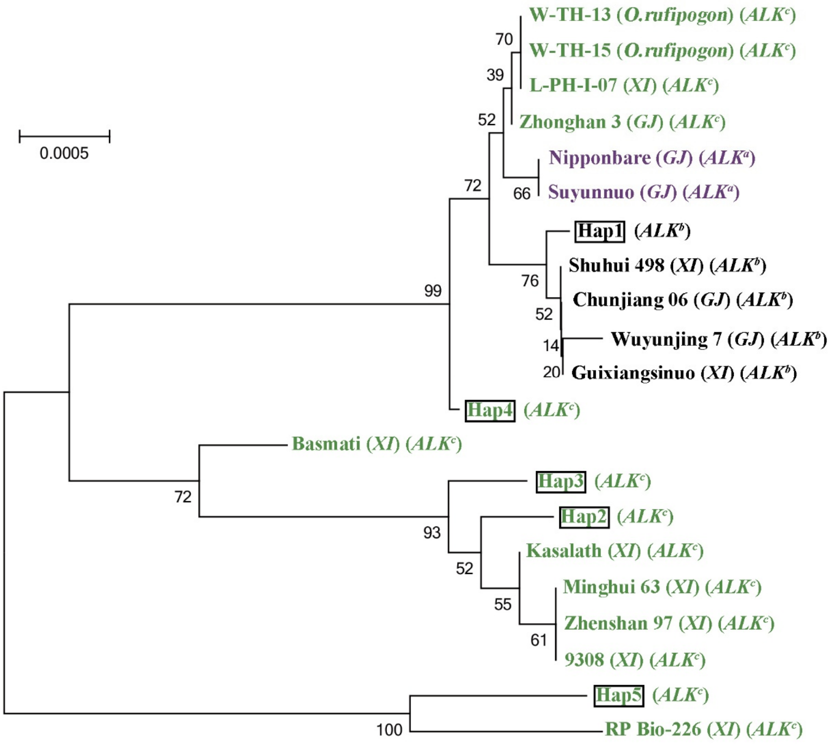

Figure S6. Clustering analysis of different rice varieties *ALK*.

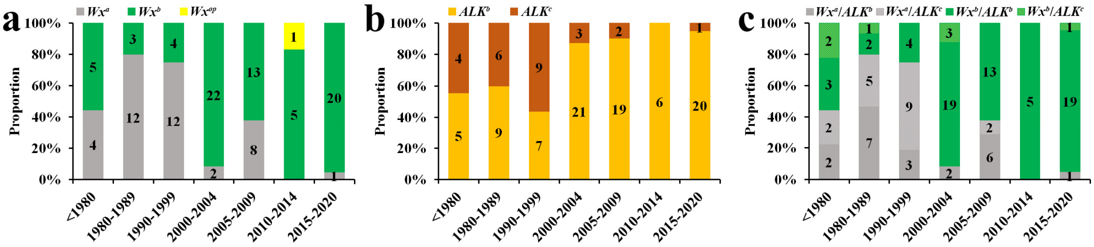

Figure S7. Frequency distribution of different rice materials based on different alleles. **(a)** *Waxy*. **(b)** *ALK*. **(c)** Combination of *Wx* and *ALK*.

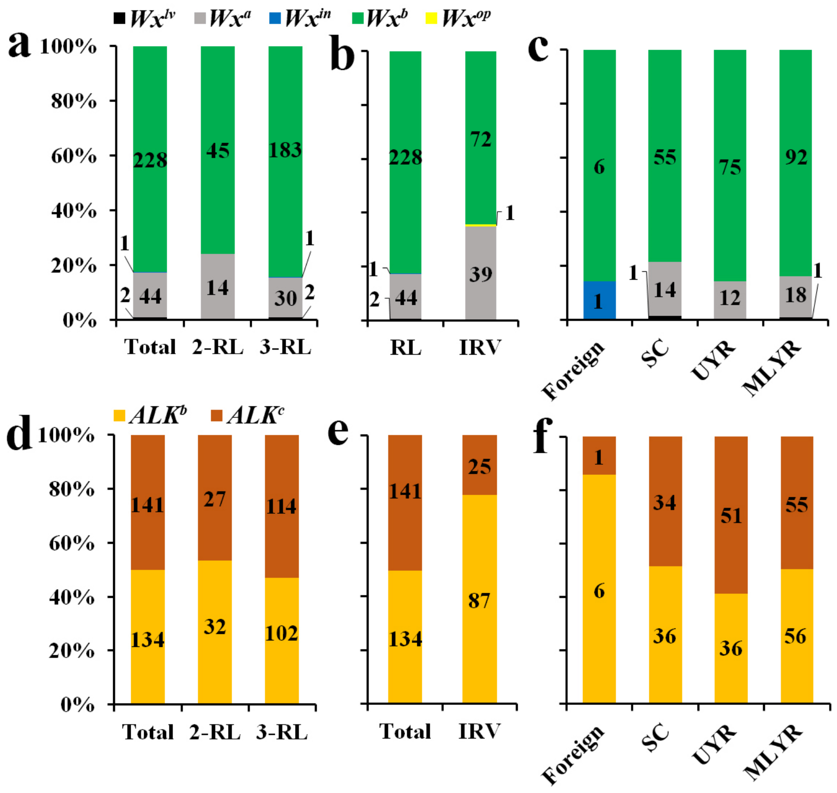

Figure S8. Frequency distribution of different rice materials based on different alleles of different types and areas. **(a, b, c)** *Waxy*. **(d, e, f)** *ALK*.

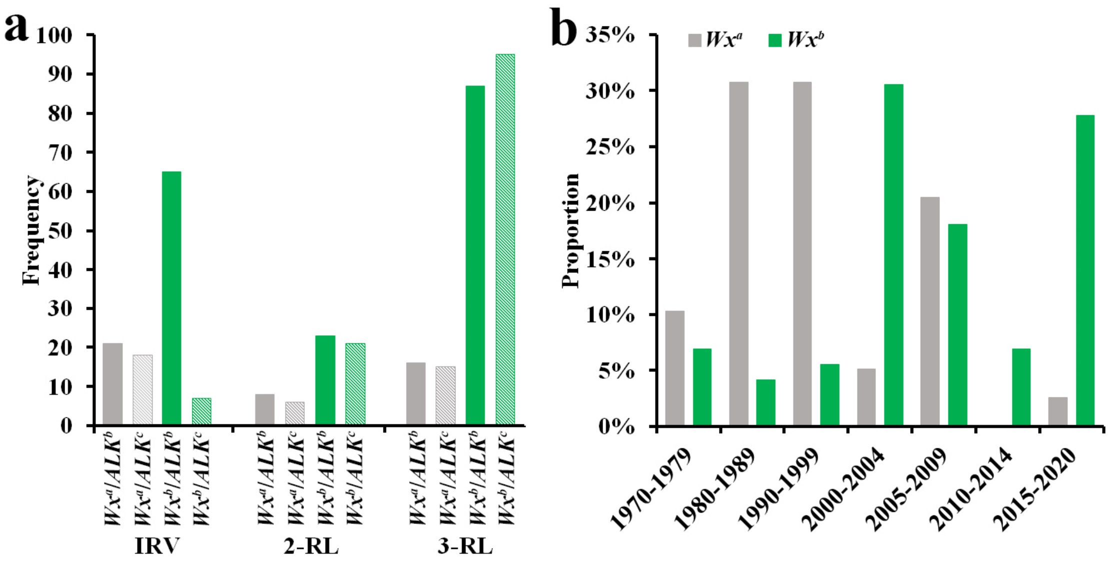

Figure S9. Combined analysis of *Waxy* and *ALK*. **(a)** Frequency distribution of different combinations. **(b)** Distribution of Wx^a and Wx^b at different breeding times.

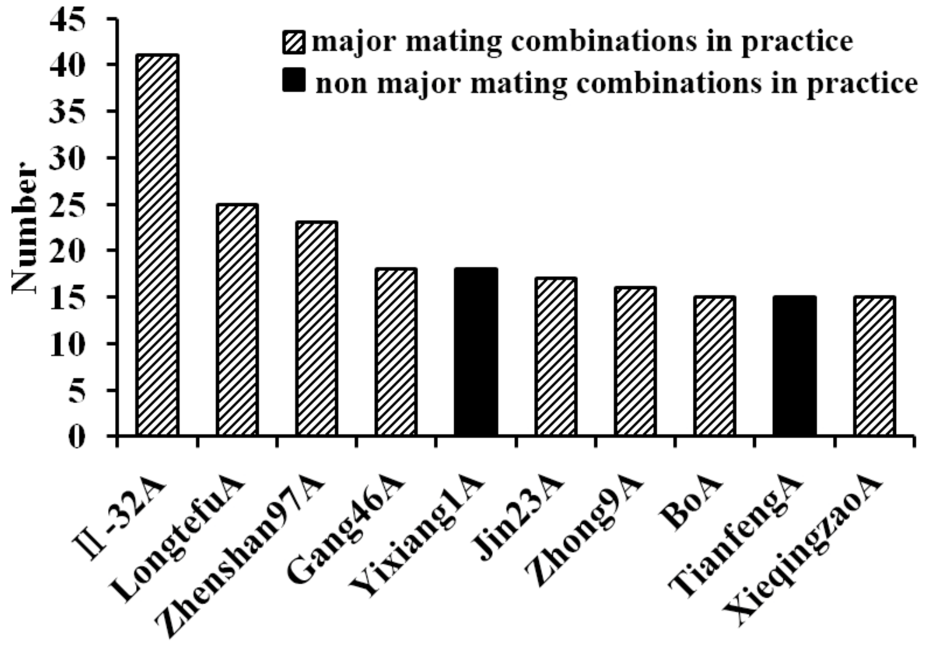

Figure S10. Female plants matching RLs in this experiment based on statistics or sterile lines.

Supplementary Tables

Table S2. Correlation analysis

0	AAC	ΔH	T_0	T_p	T_e	Taste value	Hardness	Stickiness	PKV	HPV	DBV	CPV
ΔH	-0.242											
T_0	-0.273	0.830**										
T_p	-0.301**	0.819**	0.978**									
T_e	-0.288	0.807**	0.958**	0.985**								
Taste value	-0.515**	0.180	0.195	0.219	0.232							
Hardness	0.624**	-0.179	-0.219	-0.249	-0.249	-0.904**						
Stickiness	-0.461**	0.129	0.136	0.159	0.175	0.920**	-0.929**					
PKV	-0.536**	0.261	0.296	0.290	0.262	0.406**	-0.487**	0.403**				
HPV	0.553**	-0.354**	-0.430**	-0.457**	-0.457**	-0.475**	0.503**	-0.394**	-0.054			
DBV	-0.769**	0.420**	0.502**	0.517**	0.498**	0.610**	-0.682**	0.545**	0.720**	-0.710**		
CPV	0.692**	-0.407**	-0.462**	-0.495**	-0.488**	-0.527**	0.588**	-0.465**	-0.255	0.890**	-0.801**	
SBV	0.788**	-0.433**	-0.491**	-0.511**	-0.492**	-0.592**	0.679**	-0.546**	-0.720**	0.668**	-0.962**	0.855**

Student's *t* test, significantly different at *p <0.05, **p <0.01.

Table S3. Genetic distance between different materials

	9308	Minghui63	RP-Bio-226	Zhonghan3	Nipponbare	Shuhui498	Chunjiang06	W-TH-13	Suyunuo	Basmati	Guixiangsinuo	W-TH-15	Wuyunjing7	Kasalath	L-PH-I-07	Zhenshan97	Hap1	Hap2	Hap3	Hap4
Minghui63	0.00000																			
RP-Bio-226	0.00564	0.00564																		
Zhonghan3	0.00504	0.00504	0.00585																	
Nipponbare	0.00524	0.00524	0.00605	0.00020																
Shuhui498	0.00544	0.00544	0.00625	0.00040	0.00060															
Chunjiang06	0.00544	0.00544	0.00625	0.00040	0.00060	0.00000														
W-TH-13	0.00582	0.00582	0.00670	0.00000	0.00029	0.00058	0.00058													
Suyunuo	0.00524	0.00524	0.00605	0.00020	0.00000	0.00060	0.00060	0.00029												
Basmati	0.00231	0.00231	0.00551	0.00347	0.00376	0.00405	0.00405	0.00349	0.00376											
Guixiangsinuo	0.00544	0.00544	0.00625	0.00040	0.00060	0.00000	0.00000	0.00058	0.00060	0.00405										
W-TH-15	0.00580	0.00580	0.00668	0.00000	0.00029	0.00058	0.00058	0.00000	0.00029	0.00348	0.00058									
Wuyunjing7	0.00564	0.00564	0.00646	0.00060	0.00080	0.00020	0.00020	0.00087	0.00080	0.00434	0.00020	0.00087								
Kasalath	0.00020	0.00020	0.00544	0.00483	0.00504	0.00524	0.00524	0.00553	0.00504	0.00202	0.00524	0.00551	0.00544							
L-PH-I-07	0.00580	0.00580	0.00668	0.00000	0.00029	0.00058	0.00058	0.00000	0.00029	0.00347	0.00058	0.00000	0.00087	0.00551						
Zhenshan97	0.00000	0.00000	0.00564	0.00504	0.00524	0.00544	0.00544	0.00582	0.00524	0.00231	0.00544	0.00580	0.00564	0.00020	0.00580					
Hap1	0.00532	0.00532	0.00656	0.00061	0.00082	0.00020	0.00020	0.00058	0.00082	0.00406	0.00020	0.00058	0.00041	0.00511	0.00058	0.00532				
Hap2	0.00061	0.00061	0.00594	0.00491	0.00511	0.00532	0.00532	0.00554	0.00511	0.00319	0.00532	0.00552	0.00553	0.00082	0.00552	0.00061	0.00511			
Hap3	0.00082	0.00082	0.00553	0.00491	0.00512	0.00532	0.00532	0.00554	0.00512	0.00203	0.00532	0.00552	0.00553	0.00061	0.00552	0.00082	0.00512	0.00122		
Hap4	0.00471	0.00471	0.00594	0.00041	0.00061	0.00082	0.00082	0.00029	0.00061	0.00319	0.00082	0.00029	0.00102	0.00450	0.00029	0.00471	0.00061	0.00491	0.00450	
Hap5	0.00574	0.00574	0.00204	0.00594	0.00615	0.00636	0.00636	0.00613	0.00615	0.00552	0.00636	0.00612	0.00656	0.00553	0.00611	0.00574	0.00615	0.00553	0.00533	0.00595

Table S4. Primers for KASP genotyping of different alleles of *Waxy* and *ALK*

KASP marker	Allele	Polymorphic site	Primer name	Primer sequence (5′–3′)
Wx-SNP1	C/T	In1-1	*Wx*-SNP1-Fam	gaaggtgaccaagttcatgctTCATCAGGAAGAACATCTGCAAGG
			Wx-SNP1-Hex	gaaggtcggagtcaacggattTCATCAGGAAGAACATCTGCAAGT
			Wx-SNP1_Com	GCCCAACACCTTACAGAAATTAGCA
Wx-SNP2	G/23bp	Ex2-112	*Wx*-SNP2-Fam	gaaggtgaccaagttcatgctTTCCAGGGCCTCAAGCCCC
			Wx-SNP2-Hex	gaaggtcggagtcaacggattGTTCCAGGGCCTCAAGCCCA
			Wx-SNP2-Com	CGCTGGTCGTCACGCTGA
Wx-SNP3	G/A	Ex4-53	*Wx*-SNP3-Fam	gaaggtgaccaagttcatgctGAGGTTTTTCCATTGCTACAAGCG
			Wx-SNP3-Hex	gaaggtcggagtcaacggattGAGGTTTTTCCATTGCTACAAGCA
			Wx-SNP3-Com	TAATGATGACTCCACCTTCTCCAG
Wx-SNP4	A/G	Ex4-77	*Wx*-SNP4-Fam	gaaggtgaccaagttcatgctGTCGACCGTGTGTTCATCGA
			Wx-SNP4-Hex	gaaggtcggagtcaacggattGTCGACCGTGTGTTCATCGG
			Wx-SNP4-Com	CAAACCTGAAATCACCAGTGGAAG
Wx-SNP5	A/C	Ex6-62	*Wx*-SNP5-Fam	gaaggtgaccaagttcatgctTCTTGAGATCAATTGTAACTCACCAT
			Wx-SNP5-Hex	gaaggtcggagtcaacggattTCTTGAGATCAATTGTAACTCACCAG
			Wx-SNP5-Com	GCTCCTAGGATCCTAAACCTCAAC
Wx-SNP6	C/T	Ex10-115	*Wx*-SNP6-Fam	gaaggtgaccaagttcatgctCTGGAGGAACAGAAGGGCC
			Wx-SNP6-Hex	gaaggtcggagtcaacggattCTGGAGGAACAGAAGGGCT
			Wx-SNP6-Com	GAAGAACGATCTGGACGTCCTC
ALK-SNP1	A/G	Ex8-733	ALK-SNP2-Fam	gaaggtgaccaagttcatgctGAACGGGTCGAACGCCGACAT
			ALK-SNP2-Hex	gaaggtcggagtcaacggattACGGGTCGAACGCCGACAC
			ALK-SNP2-Com	GTCGGCGGGCTGAGGGACA
ALK-SNP2	GC/TT	Ex8-864/865	ALK-SNP4-Fam	gaaggtgaccaagttcatgctGACATGCCGCGCACCTGGAA
			ALK-SNP4-Hex	gaaggtcggagtcaacggattACATGCCGCGCACCTGGAG
			ALK-SNP4-Com	GCCTCGAGACGTACCGCAAGTA
